# Unilateral primary aldosteronism considerations about the diagnostic criteria, adrenalectomy, and short and long time biochemical and clinical evaluation

**DOI:** 10.1111/jch.14394

**Published:** 2021-11-30

**Authors:** Decio Armanini, Marco Boscaro, Chiara Sabbadin, Luciana Bordin

**Affiliations:** ^1^ Department of Medicine‐Endocrinology Università degli Studi di Padova Padova Italy; ^2^ Department of Molecular Medicine‐Biological Chemistry Università degli Studi di Padova Padova Italy

## 1 GENERAL CONSIDERATIONS

The study by Yan and co‐workers^1^ sought to review the literature reports on diagnostic criteria and biochemical and clinical success after surgery in patients with unilateral hyperaldosteronism. Primary aldosteronism is a syndrome, that includes various pathologies that have certain signs and symptoms in common. Since the discovery of the disease, many studies have tried to establish the criteria for diagnosis and operability of unilateral forms. With the availability of computerized tomography (CT) and magnetic resonance (MRI) and with the advance in methods to evaluate selective aldosterone secretion in the adrenal veins (AVS), the criteria have been refined and numerous guidelines have been made by various societies. The initial guidelines of the Endocrine Society and the Japan Endocrine Society^2^, ^3^ recommend AVS in all patients diagnosed with primary aldosteronism who are willing to have surgery when necessary. Subsequently, new guidelines considered that in some cases catheterization was not necessary if an adenoma was evident in a young patient with low serum potassium.^4^ The modification of the guidelines demonstrates that many points remain unclear and may influence the follow‐up after unilateral adrenalectomy. The difficulty to create homogeneous guidelines is probably related to the fact that primary aldosteronism is a syndrome and not a defined disease with numerous underlying factors (genetic, autoimmune, family, etc.).^5^

The Primary Aldosteronism Surgical Outcome (PASO) study^6^ emphasized that the duration of hypertension is a key factor in both the outcome of the intervention of future cardiovascular risk. Establishing the duration of hypertension is not always easy. We are dealing with hypertensive patients at diagnosis and sometimes it is difficult to establish the duration of hypertension, considering that not everyone has had their blood pressure measured and the aldosterone to renin ratio evaluated before they first noticed blood pressure was high. This point is important because we must always consider that increased aldosterone can cause permanent damage to the arteries and metabolic complications, and thus be a negative prognostic factor after unilateral adrenalectomy. It is true that the aldosterone to renin ratio often normalizes, but possible vascular damage and cardiovascular risk is a consequence of both the direct effect of aldosterone at the level of vascular wall and, in a lesser extent, of any other cause of hypertension. It is still unclear whether PA arises ex novo on a normotensive patient or overlaps in an already hypertensive patient. Recently, aldosterone has been shown to be the major proinflammatory and profibrotic hormone, and this must be considered in both the collection of the patient's history and the therapy to be performed

The publication of Yan and collaborators^1^ considered the literature studies performed to assess the biochemical and clinical effect of AVS or CT, evaluating the result of the surgery. The accuracy of diagnosis by imaging tests such as CT or MRI are not always precise, so all guidelines have recommended AVS, as morphological examinations are unable to detect small adenomas. The differential diagnosis between unilateral and bilateral forms has always been critical. A recent meta‐analysis evaluated the accuracy of adrenal imaging examinations for the evaluation of unilateral forms, considering the result of AVS. The analysis ascertained a sensitivity of 68% and specificity of 57% for CT/MRI in identifying unilateral forms The parameters were higher considering patients with age < 40 years (71 and 79%). The study concluded that CT/MRI are not efficient tests as an alternative to AVS even in young patients,^7^ but the literature studies are non‐homogeneous.^8^, ^9^ An important consideration in the evaluation of the studies in the literature is that usually CT/MRI and AVS are performed in the same patient and sometimes the decision to do AVS is made just based on the CT image.

AVS can sometimes give problems of interpretation both for the difficulty of cannulating the right adrenal vein and for the presence of anatomical variants of the adrenal veins that can dilute the blood. Other problems may be stress‐related particularly in situations of technical difficulty during catheterization. Stress evoked by the procedure may activate ACTH, cortisol, and aldosterone, interfering with lateralization. Also, the pulsatile pattern of secretion of cortisol and aldosterone can generate time‐related variability in hormone concentrations in the adrenal vein blood. For this reason, some authors prefer to associate ACTH stimulation to minimize the time‐related variability compared with sequential sampling without stimulation.^10^

These factors and the evaluation of other clinical parameters must be considered to decide on the intervention and to predict its success. Resolution of hypertension occurs in only 30–60% of cases.^1^ Chronic therapy with aldosterone receptor blockers alone or with other hypotensive agents is recommended if the patient refuses surgery or if there is a risk of surgery (eg, advanced age or associated diseases) or if the patient has already had surgery. Conversely, medical management with spironolactone or eplerenone is an alternative treatment of cases of primary aldosteronism, so the choice of medical management should always be considered.

Recent guidelines recommend doing the surgery directly in young patients with a marked hypokalemia, but we must always keep in mind the possibility of non‐secreting incidentalomas in patients not treated before surgery with aldosterone receptor blockers.^11^

Most of the centers prefer to perform surgery even in cases where the CT scan does not show an adenoma and the AVS shows lateralization, but in these cases, we believe to be preferable to perform the therapy with aldosterone receptors blockers and reserve another AVS later especially if the patient is not well controlled with the therapy. Given that in these cases hypertension often persists even after surgery, sometimes associated with persistent increase of aldosterone to renin ratio, it would be more appropriate to first treat the patient with anti‐aldosterone therapy and then reconsider the possibility of surgery.

Normalization of blood pressure and serum potassium during aldosterone receptor blockers before surgery can sometimes be a criterion for the presumption of complete clinical and biochemical recovery.

Both the persistence of hyperaldosteronism due to incorrect interpretation of biochemical and morphological tests, and the persistence of hypertension even in the presence of resolution of primary aldosteronism should be evaluated in relation to the future risk not only of cardiovascular accidents, but also metabolic ones, such as diabetes.

The authors have reported correctly that the prognosis after adrenalectomy guided by CT versus AVS is heterogeneous consistent with the opportunity of a more accurate assessment prior to intervention with presumption of both clinical and biochemical success. Therapy including anti‐aldosterone drugs even in some cases not operated could be appropriate considering the classic Pitt studies^12^ that have shown that such therapy protects from the risk of cardiovascular relapse regardless of the pressure and aldosterone values. It is known that aldosterone is a factor that exacerbates cardiovascular risk because of its proinflammatory and profibrotic actions.^13^ Hundemer and collaborators^14^ showed that cardiovascular risk is greater in patients treated with mineralocorticoid receptor blockers compared with essential hypertensive patients treated with conventional therapies. Considering the Pitt's studies^12^ probably the risk is dependent on the duration of hypertension and should also take in mind the much greater risk that would have occurred if patients had not been treated with aldosterone receptor blockers. An important factor to be considered will also be the direct action of spironolactone at the adrenal level of blocking aldosterone synthase. A similar effect after long‐term treatment with aldosterone receptors blockers in patients with primary aldosteronism could lead to a restoration of proper functioning of the renin‐angiotensin‐aldosterone system as demonstrated in cases of idiopathic hyperaldosteronism that have recovered from the condition after prolonged treatment with anti‐aldosteronics.^15^

## 2 CONCLUSIVE REMARKS

Clinical and biochemical success is not a sufficient criterion to understand the future of these patients, as pointed in the reference study.^1^ Most of the guidelines have focused on biochemical or clinical parameters assessment after surgery, but future studies will need to assess subsequent long‐term cardiovascular risk comparing the long‐term effects of adrenalectomy and those of chronic therapy with aldosterone receptors blockers combined with other hypotensive agents. These studies should consider that about 60% of operated patients are hypertensive after surgery^1^ and that sometimes primary aldosteronism can persist even after surgery in cases that were not correctly evaluated.

## FUNDING

The authors declare that there are no funders for this work.

## CONFLICT OF INTEREST

The authors have no competing interests.
